# Identifying emphysema risk using brominated flame retardants exposure: a machine learning predictive model based on the SHAP methodology

**DOI:** 10.3389/fpubh.2025.1600729

**Published:** 2025-06-25

**Authors:** Qihang Xie, Haoran Qu, Jianfeng Li, Rui Zeng, Wenhao Li, Rui Ouyang, Chengxiang Zhang, Siyu Xie, Ming Du

**Affiliations:** ^1^Department of Cardiothoracic Surgery, The First Affiliated Hospital of Chongqing Medical University, Chongqing, China; ^2^Department of General Medicine, The First Affiliated Hospital of Chongqing Medical University, Chongqing, China; ^3^Department of Child Development, United Graduate School of Child Development, Osaka University, Suita, Osaka, Japan; ^4^United Graduate School of Child Development, The University of Osaka 2-2 Yamadaoka, Suita, Osaka, Japan

**Keywords:** machine learning, SHAP, environmental exposure, brominated flame retardants, emphysema

## Abstract

**Background:**

Emphysema is a major contributor to lung disease progression and is associated with significant health risks, including exacerbations, mortality, and lung cancer. While environmental exposures, such as brominated flame retardants (BFRs), have been suggested as risk factors, their role in emphysema prediction has been largely overlooked. This study aimed to develop a machine learning (ML) model to predict emphysema risk incorporating BFRs exposure data and demographic characteristics.

**Methods:**

Using data from the NHANES (2005–2016) dataset, 8,205 participants were included in the study. The participants were divided into a training set (70%) and a testing set (30%). Eight machine learning algorithms, including lightGBM, MLP, DT, KNN, RF, SVM, Enet, and XGBoost, were applied to build and evaluate the model. Demographic data and BFRs exposure levels were used as predictors. SHAP and Partial Dependence Plots (PDP) were used for model interpretability analysis.

**Results:**

The MLP model showed the best performance with an AUC of 0.83. Age and PBB153 were identified as the most influential predictors. SHAP analysis revealed that higher exposure to BFRs, particularly PBB153, was strongly associated with increased emphysema risk. The WQS model further confirmed the positive relationship between BFRs exposure and emphysema.

**Conclusion:**

This study demonstrates the significant predictive value of BFR exposure in emphysema risk assessment and highlights the importance of incorporating environmental factors into disease prediction models. The findings provide new insights for integrating BFRs into personalized health risk assessments and public health interventions.

## 1 Introduction

Emphysema, a chronic respiratory disorder defined by alveolar destruction, is a significant phenotype of COPD (1). Patients exhibiting more severe emphysema experience accelerated decline in lung function, body mass index, and fat-free mass index, accompanied by increased exacerbations, hospitalizations, and mortality rates (2). The efficacy of current treatment regimens is diminished in patients with emphysema associated with COPD (1). Emphysema serves as a substantial predictor of lung cancer risk and overall health outcomes. Studies have demonstrated that individuals with radiographic emphysema exhibit nearly double the incidence of lung cancer compared to those without (3). Given the elevated risk of exacerbations, mortality, and lung cancer associated with emphysema (4), its early identification through screening and advanced prediction methods could significantly improve patient outcomes and guide preventive interventions.

In previous studies, scholars employed blood-based emphysema predictive models that exhibited two notable limitations. Firstly, these models had relatively small sample sizes, which can compromise their ability to accurately predict outcomes. Secondly, they were limited in their scope, as they only tested one “omic” modality at a time (5–7). Other studies have used transcriptomic and proteomic features in combination with clinical features to evaluate the role of multiomics modeling in predicting emphysema. Ultimately, the best-performing predictive model (clinical + CBC + protein model) included predictors of clinical variables (age, sex, ethnicity, BMI, smoking), CBC (proportion of neutrophils, lymphocytes, platelets, monocytes, and eosinophils), and protein. In the clinical + CBC + gene + protein model, the top 10 predictors were ranked by absolute *β* coefficient, including BMI, sRAGE, PSMP protein and MIR124-1HG gene (8). There are also CT image-based models for predicting the progression of emphysema (9). However, none of them paid attention to the influence of environmental factors on the disease of emphysema.

Brominated flame retardants (BFRs) are utilized extensively in various industrial sectors, including plastics, textiles, electronics, and building materials, with the primary objective of mitigating the risk of fire hazards (10, 11). However, as additive compounds, some BFRs, including PBDEs and TBBPA, are prone to environmental release during production and use. The presence of these chemicals has been detected in various environmental media, including water, soil, dust, and even human biological fluids such as blood and breast milk (11, 12). BFRs are persistent in the environment and can accumulate in living organisms over time.

As has been demonstrated in prior studies, BFRs and their metabolites has the potential to induce a number of deleterious effects on bodily functions, including nephrotoxicity, hepatotoxicity, reproductive and developmental toxicity, neurotoxicity, and carcinogenic effects, which can ultimately result in severe adverse health consequences (13). In more detail, the presence of BFRs has been detected in the respiratory tracts of both animals and humans. In these locations, BFRs have been shown to affect bronchial epithelial cells by means of inhibiting cell viability, activating apoptosis, inducing DNA damage, and promoting inflammatory and oxidative stress responses (14–17). These changes in the respiratory tract are significant, as they are known to play a key role in the development of emphysema (18, 19). Therefore, this study combined BFRs with demographic characteristics to construct a machine learning prediction model and determined the predictive value of BFRs exposure for emphysema.

## 2 Methods

### 2.1 Study population

**Figure 1:**
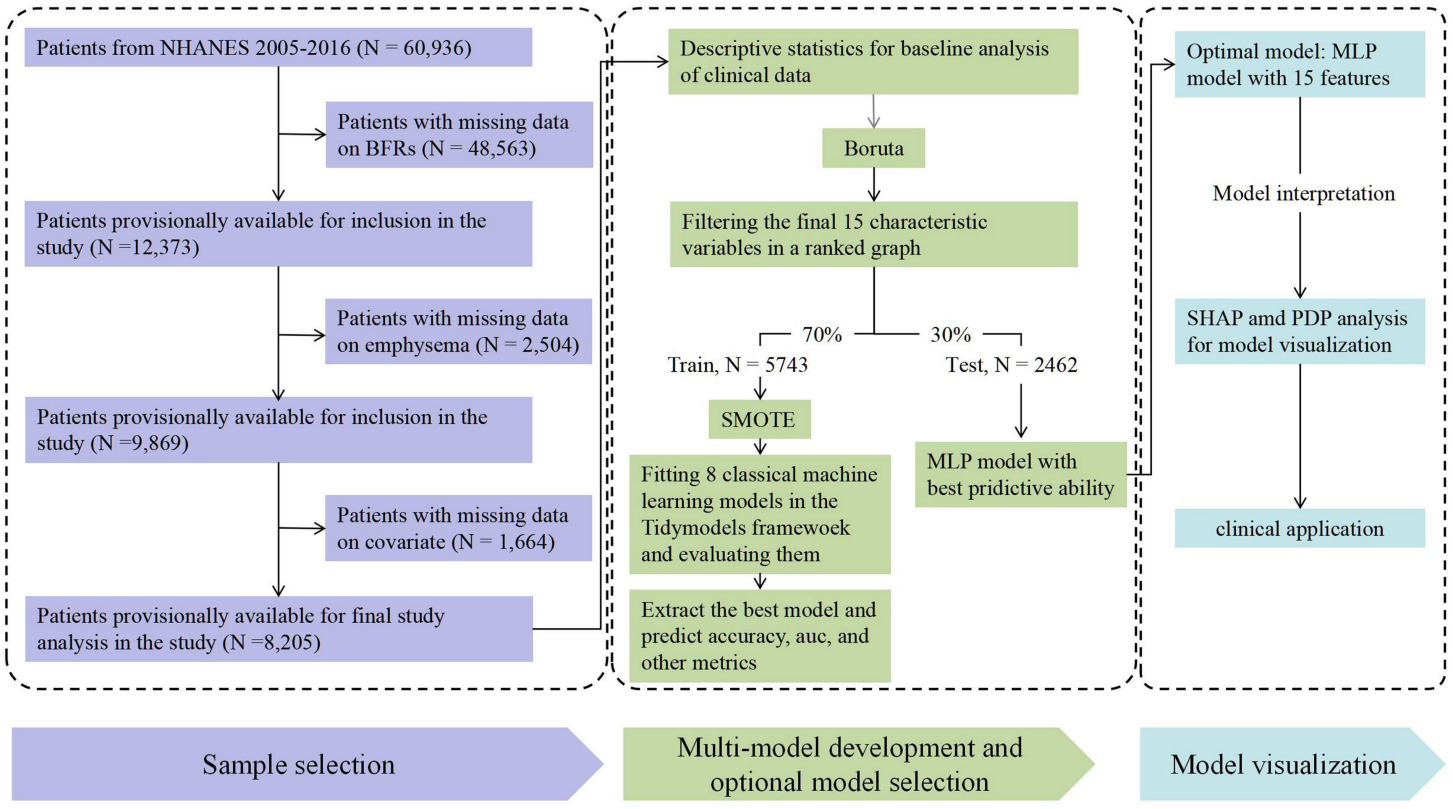
Flow chart for model development and validation.

The National Health and Nutrition Examination Survey (NHANES) is a comprehensive interdisciplinary research initiative spearheaded by the Centers for Disease Control and Prevention (CDC). The primary objective of NHANES is the collection, analysis, and publication of data concerning health, nutrition, and environmental exposures in the United States. Since its inception in the 1960s, it has been conducted on an annual basis and encompasses all age groups within the United States. In the present study, the participants from the NHANES from 2005 to 2016 with available data on BFRs were included. Participants with missing BFRs data, a diagnosis of emphysema, and covariates were excluded from the study. The inclusion and exclusion criteria utilized in this study are illustrated in Figure 1. To evaluate the potential impact of selection bias due to missing BFR data, we compared the weighted baseline characteristics between included and excluded participants.

### 2.2 Assessment of BFR

In the NHANES database, concentrations of polybrominated diphenylethers (PBDEs) in serum were assessed employing a two-phase protocol encompassing automated liquid–liquid extraction and subsequent sample purification (NHANES, 2019). Serum concentrations of BFRs were measured as parent compounds. Metabolites were not included unless specifically reported in the dataset, and we restricted our analysis to parent compounds to ensure consistency. However, in this study, we exclusively focused on PBB-153 and nine PBDEs with a detection rate greater than 50% (20). Specifically, these include 2,4,4′-tribromodiphenyl ether (BDE-28), 2,2′,4,4′-tetrabromodiphenyl ether (BDE-47), 2,2′,3,4,4′-pentabromodiphenyl ether (BDE-85), 2,2′,4,4′, 5-pentabromodiphenyl ether (BDE-99), 2,2′,4,4′,6-pentabromodiphenyl ether (BDE-100), 2,2′,4,4′,5,5′-hexabromodiphenyl ether (BDE-153), 2, 2′,4,4′,5,6′-hexabromodiphenyl ether (BDE-154), 2,2′,3,4,4′,5′,6-heptabromodiphenyl ether (BDE-183), decabromodiphenyl ether (BDE-209), and 2,2′,4,4′,5,5′-hexabromobiphenyl (PBB-153). For values below the limit of quantification (LOQ), NHANES substitutes these with LOQ divided by the square root of 2 (LOQ/√2), in line with standard imputation practices. We applied this substitution consistently across all BFR congeners to maintain comparability and avoid data loss.

### 2.3 Definition of emphysema

The emphysema status of the participants was determined according to the variable MCQ160G in the questionnaire data of NHANES 2005–2016. Individuals who responded in the affirmative to the question “Ever told you had emphysema” were classified as patients with emphysema.

### 2.4 Covariates

The present study took into consideration a number of sociodemographic characteristics, as previously established in preceding research. The characteristics in question encompassed age, gender, race, educational level, poverty income ratio (PIR), smoking and drinking status, and body mass index (BMI) (21, 22). Race was categorized as Mexican American, Other Hispanic, Non-Hispanic White, Non-Hispanic Black, Other Race – Including Multi-Racial. Education level was less than high school, high school, or college or above. PIR measured socioeconomic status as the ratio of household income to poverty line. Drinking status was coded as never, mild/moderate, heavy or former drinker categories. Smoking status included never, former and now smoker.

### 2.5 Statistical analysis

Baseline characteristics were first compared between the training and test datasets in the NHANES. Then, within both the training and test datasets, we compared the baseline characteristics between the emphysema and non-emphysema groups. Continuous variables were presented as median (IQR) or mean (SD), and categorical variables as absolute numbers with associated percentages. The demographic characteristics of subjects with different emphysema statuses were evaluated using the chi-square test and *t*-test. Serum BFRs were Ln transformed to ensure the attainment of a near-normal distribution (continuous variables) or segmented into four quartiles (Q1, Q2, Q3, and Q4) to form categorical variables. To justify this transformation, we have included histograms comparing the distributions of BFR variables before and after Ln transformation in the Supplementary Figure 1. In order to ascertain the relationships among the concentrations of the ten BFRs, Pearson’s correlation was implemented as a statistical analysis tool. Principal component analysis (PCA) was employed to elucidate the disparities in subject composition among varying concentrations and to ascertain the underlying structure of subject variance. The Mann–Whitney U test was further used to compare the scores of the two groups on PC1 to see if there were significant differences in PC1 between different disease states.

To assess the correlation between BFRs and the incidence of emphysema, we employed univariate and multivariate logistic regression models. Odds ratios (OR) and the corresponding 95% confidence intervals (CI) were employed to identify trends in the correlations. The regression models were structured as follows: model 1 was not adjusted for any variable, while model 2 was adjusted for age, gender, race, education level, PIR, smoking status, drinking status, and BMI. To address the risk of false positives from testing 10 BFRs across two models and four quartiles, we applied False Discovery Rate (FDR) correction. To evaluate the potential impact of unmeasured confounders on the relationship between BFRs exposure and emphysema risk, we calculated the E-value through an online website^1^ (23, 24). The E-value offers a quantitative assessment of the strength of association that an unobserved confounder would need to have in order to fully nullify the observed relationship (25). As part of the sensitivity analyses, we excluded participants who had been diagnosed with emphysema within 2 years prior to the NHANES survey interview. This exclusion was applied to both logistic regression and machine learning models to minimize reverse causality and assess the robustness of the observed associations between BFR exposure and emphysema risk.

We performed a Weighted Quantile Sum (WQS) analysis to evaluate both the collective and individual effects of BFRs on the prevalence of emphysema by calculating a weighted linear index and assigning appropriate weights. Bootstrapping with 1,000 iterations was applied to construct WQS indices in both positive and negative directions. When the WQS index showed statistical significance, the corresponding weights were analyzed to determine the relative contribution of each BFR within the index to emphysema prevalence. The dataset was randomly partitioned, with 40% allocated to the training set and the remaining 60% designated as the validation set.

### 2.6 Model development and comparison

All model development procedures were conducted within the tidymodels framework in R. The dataset was first randomly split into training (70%) and testing (30%) sets. To reduce the adverse impact of high-dimensional data on model performance, feature selection was performed on the training set using the Boruta algorithm. This random forest–based wrapper method identifies important predictors through iterative comparison with shadow features and is considered more stable than conventional filtering techniques (26). Although drinking and smoking were not selected by Boruta, they were retained in the model due to their well-established association with emphysema, as reported in previous studies (27, 28). Sensitivity analyses excluding these two variables were also conducted to test the robustness of findings.

After variable selection, the Synthetic Minority Oversampling Technique (SMOTE) was applied to the training data to address class imbalance between emphysema and non-emphysema participants. We then trained eight machine learning algorithms: Light Gradient Boosting Machine (LightGBM), Multi-Layer Perceptron (MLP), Decision Tree (DT), K-Nearest Neighbors (KNN), Random Forest (RF), Support Vector Machine (SVM), Elastic Net (ENet), and Extreme Gradient Boosting (XGBoost). Each model underwent hyperparameter tuning through ten-fold cross-validation within the training set to optimize performance. The full grid search space and final selected hyperparameters are detailed in eMethods. The Area Under the Receiver Operating Characteristic Curve (AUROC) was used to assess the predictive accuracy of the models during validation, with the goal of comparing the models based on their best performance. AUROC values range from 0.5 to 1.0, with higher values indicating better predictive capability. In addition to AUROC, several other performance metrics, including F1 score, precision, accuracy, recall, sensitivity, specificity, and the Matthews correlation coefficient (MCC), were also calculated to provide a comprehensive assessment of model effectiveness. To formally compare model discrimination, pairwise DeLong tests were conducted across classifiers, with False Discovery Rate (FDR) correction applied to account for multiple comparisons. To address potential optimism introduced by internal SMOTE application, we performed 100 bootstrap resampling iterations on the training dataset. Apparent AUCs, optimism estimates, and bias-corrected AUCs were calculated across iterations to provide a more realistic assessment of model performance. Model calibration was evaluated using the Brier score, calibration intercept, and calibration slope. To improve the reliability of predicted probabilities, Platt scaling was applied, and calibration metrics were compared before and after adjustment. Performance variability and calibration quality were visualized using bootstrap AUC distribution plots and calibration curves, enabling a comprehensive assessment of both discrimination and probability estimation.

### 2.7 Model interpretation

Interpretability is defined as the process of elucidating how machine learning (ML) models produce results. The opacity intrinsic to machine learning (ML) models frequently hinders their effective utilization in clinical contexts, prompting comprehensive investigation into enhancing their interpretability (29, 30). In this study, we sought to integrate an interpretable method to ascertain the importance of features and the relationships between bronchodilator-related (BFR) variables and the risk of emphysema. An in-depth evaluation was conducted to ascertain the key features that exert a substantial influence on the risk of emphysema development. This evaluation utilized two approaches: shapley additive explanations (SHAP) and partial dependence plot (PDP) (31, 32). The present study analyses non-linear relationships with PDPs, thereby enabling the identification of relationships between emphysema and its associated predictors. Specifically, one-way PDPs have the capacity to elucidate the relationship between emphysema and a specific variable (33).

In this study, we adhered to the guidelines set forth in the Transparent Reporting of a Multivariable Prediction Model for Individual Prognosis or Diagnosis (TRIPOD) to maintain transparency and methodological rigor throughout the development and validation of our predictive model. No weighted data were applied, as demographic factors were adjusted for in the analysis (34). All statistical analyses were performed using R statistical software version 4.4.3. and Python 3.11. *p* < 0.05 was considered statistically significant.

## 3 Results

### 3.1 Population characteristics

In the NHNAES database from 2005 to 2016, a total of 60,936 participants were initially included. Those with missing data on BFRs (*n* = 48,563), emphysema (*n* = 2,504), or covariate data (*n* = 1,664) were excluded from the study. Finally, our study included 8,205 participants, as shown in Figure 1. Table 1 presented the demographic features of the training and test datasets. Most features showed no significant differences between the training and test sets, indicating a relatively balanced distribution of data. However, the distribution of emphysema demonstrated a statistically significant difference (*p* = 0.015), with a slightly higher prevalence of emphysema observed in the test set compared to the training set. Table 2 presents the differences in baseline characteristics between the emphysema and non-emphysema groups within the training and test datasets. In the training dataset, there were 100 cases of emphysema, accounting for 1.75% of the total; in the test dataset, there were 63 cases of emphysema, accounting for 2.56%. Significant differences were observed between the emphysema and control groups in terms of age, race, education level, PIR, drinking status, and smoking status (all *p* < 0.05). In terms of BFRs, LBCBB1 and LBCBR9 in the training set showed significant differences between the emphysema and non-emphysema groups (*p* = 0.003 and *p* < 0.001), while in the test set, LBCBB1 and LBCBR2 exhibited statistically significant differences (*p* = 0.023 and *p* = 0.016). Pearson correlation analysis identified significant positive correlations among several BFRs (Supplementary Figure 2). Specifically, BDE28, BDE47, BDE85, BDE99, BDE100, and BDE154 demonstrated strong intercorrelations. These results indicate the possibility of shared exposure sources or similar environmental behaviors among PBDEs, potentially amplifying their cumulative effect on emphysema risk. PCA revealed the relationships between individual exposome factors and emphysema, focusing on the first two principal components (Supplementary Figure 3). The Mann–Whitney U test hinted significant differences of BFRs exposure between the emphysema and control groups (*p* = 0.008). As shown in Supplementary Table 1, participants with available BFR measurements differed significantly from those without in several characteristics. Individuals included in the BFR analysis were older (mean age: 47.2 vs. 34.9 years, *p* < 0.001), had higher education levels, and a greater proportion reported former or current smoking and alcohol consumption. They also exhibited higher BMI and PIR values (all *p* < 0.001). No significant difference was observed in gender distribution (*p* = 0.26). These differences highlight potential selection bias and were considered in the interpretation of the findings.

**Table 1:** Baseline characteristics of study in the training and test cohorts.

Participant characteristics	*N* (%) or Mean (SD)			*P*-value
	Overall	Train data	Test data	
*N*	8,205	5,743	2,462	
Age (years)	49.14 (17.77)	49.20 (17.79)	49.00 (17.74)	0.645
Gender				0.52
Male	4,017 (48.96)	2,825 (49.19)	1,192 (48.42)	
Female	4,188 (51.04)	2,918 (50.81)	1,270 (51.58)	
Race or ethnicity				0.982
Mexican American	1,285 (15.66)	901 (15.69)	384 (15.60)	
Other Hispanic	781 (9.52)	547 (9.52)	234 (9.50)	
Non-Hispanic White	3,677 (44.81)	2,583 (44.98)	1,094 (44.44)	
Non-Hispanic Black	1704 (20.77)	1,187 (20.67)	517 (21.00)	
Other Race – Including Multi-Racial	758 (9.24)	525 (9.14)	233 (9.46)	
Education level				0.711
Less than high school	2004 (24.42)	1,416 (24.66)	588 (23.88)	
High School or Equivalent	1869 (22.78)	1,310 (22.81)	559 (22.71)	
College or above	4,332 (52.80)	3,017 (52.53)	1,315 (53.41)	
PIR	2.55 (1.63)	2.55 (1.63)	2.55 (1.63)	0.924
BMI (kg/m^2^)	29.13 (6.78)	29.13 (6.83)	29.14 (6.67)	0.91
Drinking status				0.578
Never	1,171 (14.27)	818 (14.24)	353 (14.34)	
Mild, moderate	3,881 (47.30)	2,739 (47.69)	1,142 (46.39)	
Heavy	1,674 (20.40)	1,150 (20.02)	524 (21.28)	
Former	1,479 (18.03)	1,036 (18.04)	443 (17.99)	
Smoking status				0.72
Never	4,480 (54.60)	3,136 (54.61)	1,344 (54.59)	
Former	2060 (25.11)	1,453 (25.30)	607 (24.65)	
Now	1,665 (20.29)	1,154 (20.09)	511 (20.76)	
PBB153 (pg/g)	31.15 (62.41)	31.40 (65.16)	30.57 (55.47)	0.557
BDE209 (pg/g)	19.87 (29.35)	19.59 (25.87)	20.53 (36.19)	0.242
BDE28 (pg/g)	9.10 (6.64)	9.17 (6.74)	8.94 (6.41)	0.143
BDE47 (pg/g)	174.14 (168.77)	176.24 (174.10)	169.23 (155.53)	0.071
BDE85 (pg/g)	3.88 (4.76)	3.93 (4.99)	3.77 (4.17)	0.139
BDE99 (pg/g)	38.76 (49.70)	39.32 (51.11)	37.45 (46.25)	0.103
BDE100 (pg/g)	36.07 (36.08)	36.37 (37.11)	35.47 (33.55)	0.307
BDE153 (pg/g)	72.59 (65.20)	72.48 (64.60)	72.86 (66.60)	0.814
BDE154 (pg/g)	3.52 (3.97)	3.57 (4.16)	3.40 (3.47)	0.054
BDE183 (pg/g)	2.03 (3.38)	2.02 (3.26)	2.04 (3.64)	0.79
Emphysema				0.015
No	8,042 (98.01)	5,643 (98.26)	2,399 (97.44)	
Yes	163 (1.99)	100 (1.74)	63 (2.56)	

Data are *n* (%), mean (SD). PIR, poverty to income ratio; BMI, body mass index.

**Table 2:** Baseline characteristics of participants by emphysema status within the training and test datasets.

Participant characteristics	*N* (%) or Mean (SD)					
	Train data		*P*-value	Test data		*P*-value
	Emphysema (*N* = 100)	Non-emphysema (*N* = 5,643)		Emphysema (*N* = 63)	Non-emphysema (*N* = 2,399)	
Age (years)	65.42 (12.03)	48.91 (17.74)	<0.001	64.06 (13.39)	48.61 (17.67)	<0.001
Gender			0.0755			0.097
Male	58 (58.00)	2,767 (49.03)		37 (58.73)	1,155 (48.15)	
Female	42 (42.00)	2,876 (50.97)		26 (41.27)	1,244 (51.85)	
Race or ethnicity			<0.001			0.002
Mexican American	5 (5.00)	896 (15.88)		3.00 (4.76)	381.00 (15.88)	
Other Hispanic	6 (6.00)	541 (9.59)		8.00 (12.70)	226.00 (9.42)	
Non-Hispanic White	67 (67.00)	2,516 (44.59)		41.00 (65.08)	1053.00 (43.89)	
Non-Hispanic Black	16 (16.00)	1,171 (20.75)		5.00 (7.94)	512.00 (21.34)	
Other Race – Including Multi-Racial	6 (6.00)	519 (9.20)		6.00 (9.52)	227.00 (9.46)	
Education level			<0.001			0.021
Less than high school	43 (43.00)	1,373 (24.33)		22.00 (34.92)	566.00 (23.59)	
High school or equivalent	25 (25.00)	1,285 (22.77)		18.00 (28.57)	541.00 (22.55)	
College or above	32 (32.00)	2,985 (52.90)		23.00 (36.51)	1292.00 (53.86)	
PIR	1.73 (1.22)	2.56 (1.63)	<0.001	2.190 (1.298)	2.563 (1.634)	0.025
BMI (kg/m^2^)	28.83 (7.40)	29.13 (6.82)	0.685	29.77 (6.83)	29.13 (6.67)	0.456
Drinking status			<0.001			<0.001
Never	9 (9.00)	809 (14.34)		4 (6.35)	349 (14.55)	
Mild, moderate	45 (45.00)	2,694 (47.74)		25 (39.68)	1,117 (46.56)	
Heavy	10 (10.00)	1,140 (20.20)		9 (14.29)	515 (21.47)	
Former	36 (36.00)	1,000 (17.72)		25 (39.68)	418 (17.42)	
Smoking status			<0.001			<0.001
Never	5 (5.00)	3,131 (55.48)		7 (11.11)	1,337 (55.73)	
Former	51 (51.00)	1,402 (24.84)		33 (52.38)	574 (23.93)	
Now	44 (44.00)	1,110 (19.67)		23 (36.51)	488 (20.34)	
PBB153 (pg/g)	51.53 (68.03)	31.04 (65.06)	0.003	42.06 (40.54)	30.27 (55.79)	0.023
BDE209 (pg/g)	17.50 (13.49)	19.62 (26.03)	0.126	18.21 (12.88)	20.59 (36.61)	0.181
BDE28 (pg/g)	10.31 (7.44)	9.15 (6.73)	0.119	11.16 (7.53)	8.88 (6.37)	0.016
BDE47 (pg/g)	187.88 (160.87)	176.03 (174.33)	0.464	210.52 (182.53)	168.14 (154.66)	0.066
BDE85 (pg/g)	4.25 (4.63)	3.93 (5.00)	0.486	4.78 (4.74)	3.75 (4.15)	0.085
BDE99 (pg/g)	41.49 (44.51)	39.28 (51.22)	0.622	48.19 (52.24)	37.16 (46.06)	0.095
BDE100 (pg/g)	37.25 (34.50)	36.31 (37.16)	0.785	42.47 (37.57)	35.29 (33.42)	0.131
BDE153 (pg/g)	88.70 (87.57)	72.20 (64.09)	0.06	86.75 (80.34)	72.49 (66.18)	0.16
BDE154 (pg/g)	3.57 (3.58)	3.57 (4.17)	0.996	4.28 (4.13)	3.37 (3.45)	0.082
BDE183 (pg/g)	1.66 (0.72)	2.03 (3.29)	<0.001	1.98 (1.38)	2.04 (3.68)	0.713

Data are *n* (%), mean (SD). PIR, poverty to income ratio; BMI, body mass index.

### 3.2 BFRs exposure and emphysema risk in the logistic regression model

Table 3 demonstrates that the ln-transformed PBB153 was significantly associated with an increased prevalence of emphysema. In Model I, without adjusting for covariates, the OR was 1.80 (95% CI: 1.58–2.05, *p* < 0.001). Similarly, in Model II, after adjusting for covariates, the OR was 1.32 (95% CI: 1.09–1.60, *p* = 0.005). Furthermore, a higher risk of emphysema was observed with increasing quartiles of PBB153 exposure. Specifically, individuals in the highest quartile (Q4) had a 4.8-fold higher risk of emphysema compared to those in the lowest quartile (Q1) in Model II (OR = 4.80, 95% CI: 1.59–14.52). A significant dose–response relationship between PBB153 and emphysema was identified (P for trend < 0.001). Similarly, BDE28 exhibited a comparable trend in Model I, with a significant dose–response relationship (P for trend = 0.004). Additionally, in Model I, BDE28, BDE47, BDE85, BDE99, and BDE153 were significantly positively associated with emphysema (*p* < 0.05). In the third quartile (Q3), BDE154 and BDE183 were associated with an increased risk of emphysema by 59 and 69%, respectively (BDE154: OR = 1.59, 95% CI: 1.01–2.51; BDE183: OR = 1.69, 95% CI: 1.05–2.72; all *p* < 0.05). After applying FDR correction, key associations remained statistically significant. Specifically, LnPBB153 (overall and Q4), LnBDE28 (overall and Q4), and LnBDE153 (overall) were significantly associated with emphysema (FDR-adjusted *p* < 0.05). Associations for other congeners, including LnBDE47 and LnBDE85, showed attenuated significance after correction but maintained consistent effect estimates. In the sensitivity analysis excluding participants diagnosed with emphysema within 2 years prior to the survey, the logistic regression results remained largely consistent with the primary analysis. Notably, the association between LnPBB153 (Q4) and emphysema remained statistically significant (OR = 4.45, 95% CI: 1.24–15.94, *p* = 0.022). Additional associations, such as those involving LnBDE153 and LnBDE209, showed effect estimates in the same direction as the main analysis (Supplementary Table 2).

**Table 3:** Multivariate logistic regression analysis of Ln-transformed BFRs for the prevalence of emphysema.

Variable	Model I				Model II			
	OR (95%CI)	*P* value	P* value	E-value (CI)	OR (95%CI)	*P* value	P* value	E-value (CI)
LnPBB153	**1.8 (1.58–2.05)**	**<0.001**	**0.01**	3 (2.54)	**1.32 (1.09–1.6)**	**0.005**	**0.1**	1.97 (1.4)
Q1	Reference				Reference			
Q2	**5.02 (1.71–14.7)**	**0.003**	**0.021**	9.51 (2.81)	2.04 (0.66–6.24)	0.214	0.954	3.5 (1)
Q3	**13.06 (4.72–36.19)**	**<0.001**	**0.01**	25.61 (8.91)	**3.24 (1.07–9.78)**	**0.037**	**0.463**	5.93 (1.34)
Q4	**22.74 (8.34–62.01)**	**<0.001**	**0.01**	44.97 (16.16)	**4.8 (1.59–14.52)**	**0.006**	**0.1**	9.07 (2.56)
P for trend	<0.001				<0.001			
LnBDE209	0.87 (0.64–1.19)	0.397	0.473	1.56 (1)	0.84 (0.62–1.16)	0.297	0.954	1.67 (1)
Q1	Reference				Reference			
Q2	1.05 (0.66–1.69)	0.827	0.862	1.28 (1)	1.13 (0.69–1.86)	0.63	0.954	1.51 (1)
Q3	1.49 (1–2.23)	0.053	0.126	2.34 (1)	1.42 (0.93–2.19)	0.106	0.954	2.19 (1)
Q4	0.68 (0.41–1.12)	0.127	0.214	2.3 (1)	0.68 (0.4–1.16)	0.162	0.954	2.3 (1)
P for trend	0.525				0.497			
LnBDE28	**1.52 (1.19–1.93)**	**0.001**	**0.01**	2.41 (1.67)	0.97 (0.74–1.28)	0.85	0.981	1.12 (1)
Q1	Reference				Reference			
Q2	**1.76 (1.06–2.92)**	**0.028**	0.085	2.92 (1.31)	1.33 (0.78–2.26)	0.292	0.954	1.99 (1)
Q3	**1.98 (1.2–3.25)**	**0.007**	**0.035**	3.37 (1.69)	1.21 (0.72–2.03)	0.471	0.954	1.71 (1)
Q4	**2.1 (1.29–3.44)**	**0.003**	**0.021**	3.62 (1.9)	1.03 (0.61–1.75)	0.898	0.981	1.21 (1)
P for trend	0.004				0.79			
LnBDE47	**1.28 (1.03–1.59)**	**0.024**	0.085	1.88 (1.21)	1.01 (0.8–1.27)	0.962	0.981	1.11 (1)
Q1	Reference				Reference			
Q2	1.13 (0.7–1.82)	0.626	0.68	1.51 (1)	0.86 (0.52–1.42)	0.557	0.954	1.6 (1)
Q3	1.47 (0.93–2.31)	0.097	0.187	2.3 (1)	1.11 (0.69–1.79)	0.668	0.954	1.46 (1)
Q4	1.5 (0.96–2.36)	0.077	0.165	2.37 (1)	0.91 (0.56–1.48)	0.706	0.981	1.43 (1)
P for trend	0.041				0.981			
LnBDE85	**1.28 (1.05–1.55)**	**0.013**	0.059	1.88 (1.28)	1.1 (0.9–1.36)	0.352	0.9543	1.43 (1)
Q1	Reference				Reference			
Q2	1.42 (0.88–2.3)	0.151	0.229	2.19 (1)	1.09 (0.66–1.8)	0.739	0.981	1.4 (1)
Q3	1.53 (0.95–2.45)	0.079	0.165	2.43 (1)	1.25 (0.76–2.05)	0.377	0.954	1.85 (1)
Q4	**1.7 (1.07–2.71)**	**0.024**	0.085	2.79 (1.34)	1.21 (0.74–1.98)	0.449	0.954	1.71 (1)
P for trend	0.027				0.386			
LnBDE99	**1.21 (1.01–1.46)**	**0.04**	0.108	1.71 (1.11)	1.03 (0.84–1.25)	0.793	0.981	1.21 (1)
Q1	Reference				Reference			
Q2	0.78 (0.48–1.27)	0.319	0.983	1.88 (1)	0.69 (0.41–1.14)	0.149	0.954	2.26 (1)
Q3	1.39 (0.9–2.13)	0.134	0.214	2.13 (1)	1.15 (0.73–1.81)	0.536	0.954	1.57 (1)
Q4	1.24 (0.8–1.93)	0.328	0.638	1.79 (1)	0.87 (0.54–1.38)	0.544	0.954	1.56 (1)
P for trend	0.087				0.91			
LnBDE100	1.16 (0.93–1.44)	0.187	0.267	1.59 (1)	1 (0.79–1.25)	0.979	0.981	1 (1)
Q1	Reference				Reference			
Q2	1 (0.62–1.59)	0.983	0.983	1 (1)	0.81 (0.5–1.31)	0.389	0.954	1.77 (1)
Q3	1.4 (0.9–2.15)	0.132	0.214	2.15 (1)	1.25 (0.79–1.97)	0.344	0.954	1.81 (1)
Q4	1.14 (0.72–1.79)	0.574	0.638	1.54 (1)	0.84 (0.52–1.35)	0.466	0.954	1.67 (1)
P for trend		0.309				0.915		
LnBDE153	**1.36 (1.1–1.69)**	**0.005**	**0.028**	2.06 (1.43)	1.13 (0.9–1.42)	0.283	0.954	1.51 (1)
Q1	Reference				Reference			
Q2	1.42 (0.9–2.24)	0.137	0.214	2.19 (1)	1.47 (0.9–2.38)	0.12	0.954	2.30 (1)
Q3	1.03 (0.63–1.69)	0.899	0.917	1.21 (1)	0.94 (0.56–1.58)	0.818	0.981	1.32 (1)
Q4	1.67 (1.07–2.6)	0.024	0.08	2.73 (1.34)	1.38 (0.85–2.21)	0.19	0.954	2.10 (1)
P for trend	0.074				0.487			
LnBDE154	1.18 (0.97–1.44)	0.102	0.189	1.64 (1)	0.98 (0.79–1.21)	0.832	0.981	1.16 (1)
Q1	Reference				Reference			
Q2	1.3 (0.81–2.09)	0.278	0.386	1.92 (1)	0.89 (0.55–1.47)	0.659	0.954	1.50 (1)
Q3	**1.59 (1.01–2.51)**	**0.044**	0.11	2.56 (1.11)	1.2 (0.75–1.93)	0.45	0.954	1.69 (1)
Q4	1.39 (0.87–2.21)	0.17	0.25	2.13 (1)	0.89 (0.54–1.47)	0.654	0.954	1.50 (1)
P for trend	0.12				0.962			
LnBDE183	0.95 (0.72–1.26)	0.728	0.775	1.29 (1)	0.87 (0.64–1.19)	0.386	0.954	1.56 (1)
Q1	Reference				Reference			
Q2	1.25 (0.77–2.02)	0.375	0.457	1.81 (1)	1.16 (0.69–1.93)	0.58	0.954	1.59 (1)
Q3	**1.69 (1.05–2.72)**	**0.029**	0.085	2.77 (1.28)	1.37 (0.83–2.26)	0.218	0.954	2.08 (1)
Q4	1.21 (0.73–1.99)	0.466	0.541	1.71 (1)	1.09 (0.63–1.89)	0.758	0.983	1.40 (1)
P for trend	0.299			1.37 (1)	0.636			1.24 (1)

Model 1: no covariates were adjusted. Model 2: adjusted for all covariates. P* value: adjusted for false discovery rate correction. Bold values indicate *p* < 0.05.

### 3.3 BFRs exposure and emphysema risk in WQS model

We utilized the WQS model to evaluate the association between the combined effects of BFRs and the prevalence of emphysema. As shown in Supplementary Table 3, the WQS index demonstrated a positive association between BFR exposure and emphysema prevalence (Model I: OR = 2.28, 95% CI: 1.80–2.89, *p* < 0.001; Model II: OR = 1.51, 95% CI: 1.11–2.06, *p* = 0.008). Supplementary Figure 4 illustrates that, among the BFRs, PBB153 was assigned the highest weight (0.62) in the positive direction, indicating its substantial contribution to emphysema risk after adjusting for all covariates. In contrast, the WQS regression in the negative direction did not reveal any significant association between BFR exposure and emphysema prevalence (Model I: OR = 0.93, 95% CI: 0.75–1.16, *p* = 0.522; Model II: OR = 0.86, 95% CI: 0.65–1.14, *p* = 0.291).

### 3.4 Model variable selection

**Figure 2:**
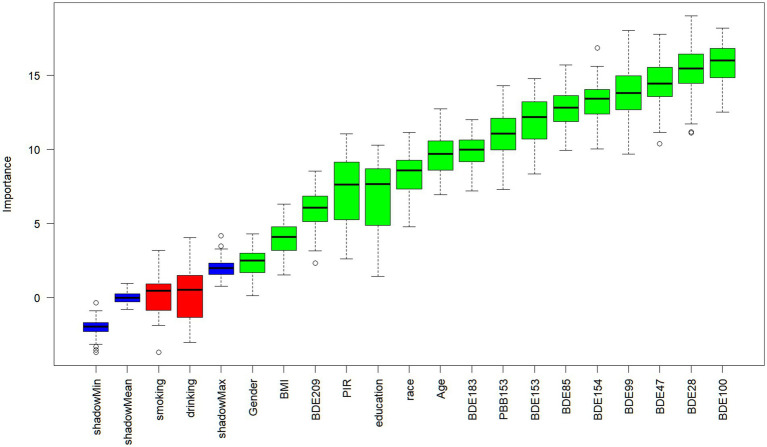
Image of Boruta method for selecting ML model variables.

Subsequently, this study identified 15 potentially significant predictor variables (highlighted as green modules in Figure 2) using the Boruta algorithm with shaded features. These selected variables, including age, gender, race, education level, PIR, BMI, PBB153, BDE28, BDE47, BDE85, BDE99, BDE100, BDE153, BDE154, BDE183, and BDE209, were utilized to train and develop the machine learning model. However, considering that previous studies have shown that smoking and alcohol consumption are important risk factors for emphysema, we included smoking and drinking as predictors in the model (27, 28).

### 3.5 Model development and performance comparison

**Figure 3:**
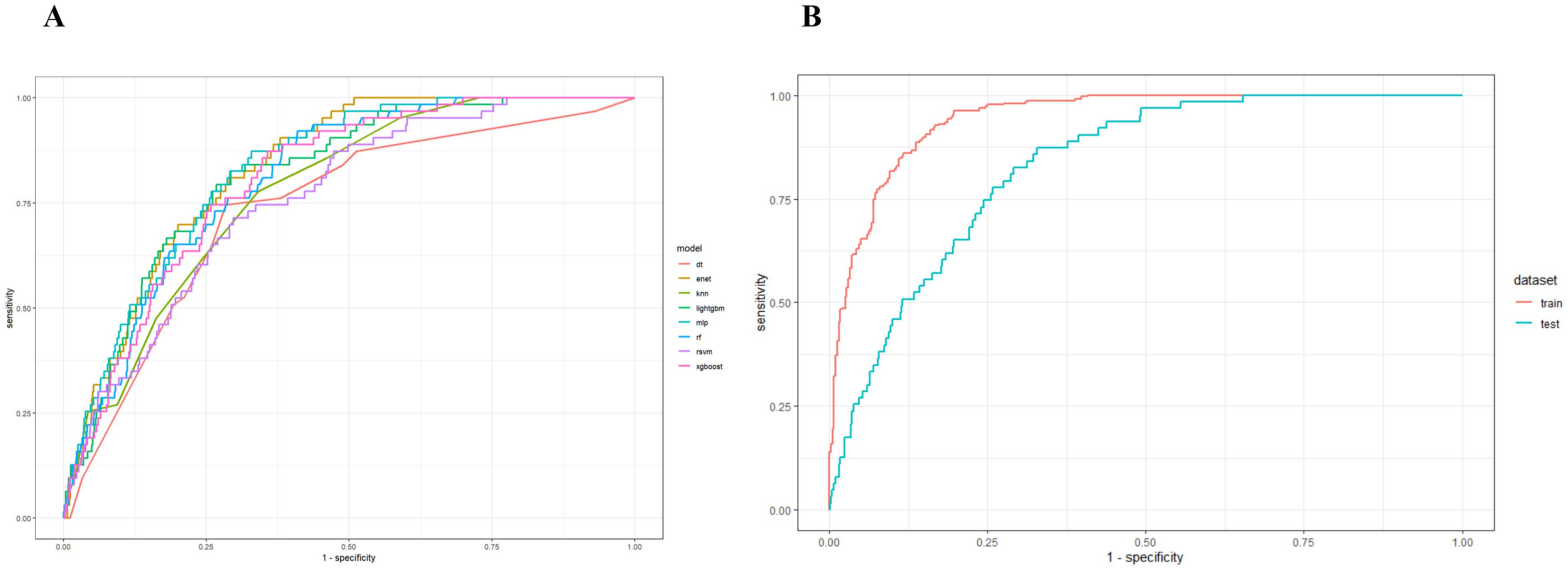
ROC curves of ML models. **(A)** ROC curves of the test sets of 8 ML models. **(B)** ROC curves of the training and test sets of MLP.

**Figure 4:**
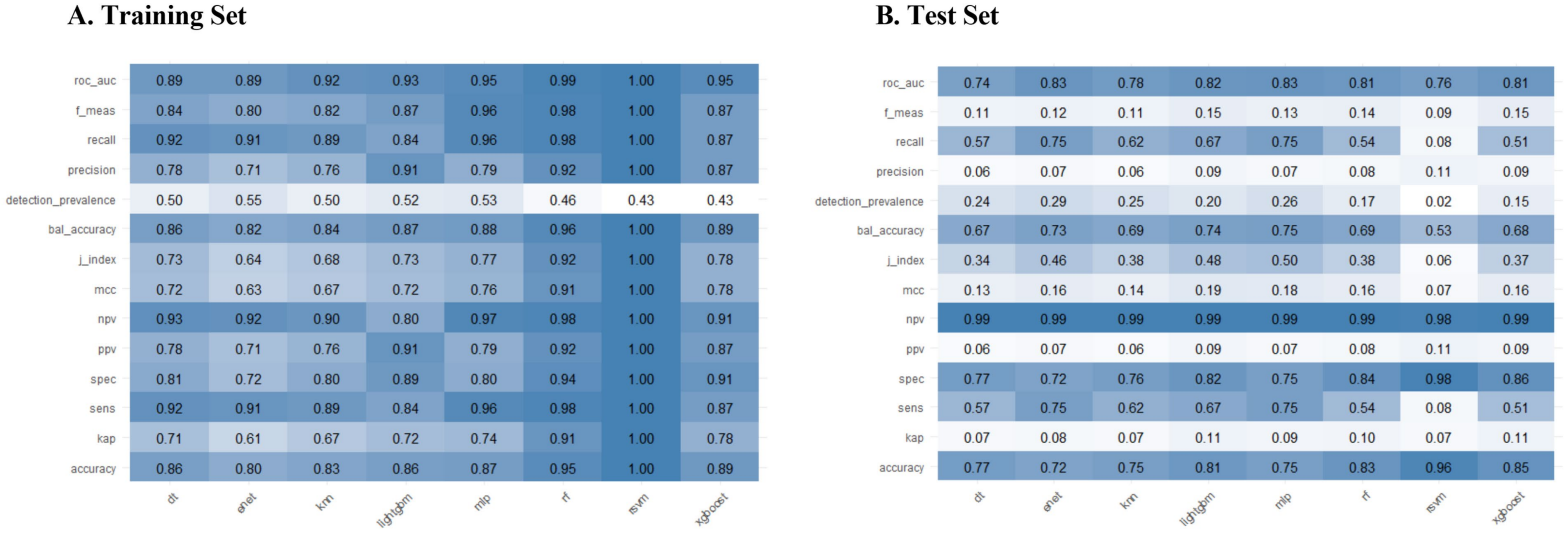
Performance comparison of various machine learning models on the training **(A)** and test **(B)** sets across multiple evaluation metrics. The heatmaps illustrate the performance of lightGBM, MLP, DT, KNN, RF, SVM, Enet, and XGBoost models. Each cell corresponds to the value of a specific evaluation metric, including accuracy, balanced accuracy, F1 score, J-index, kappa, Matthews correlation coefficient (MCC), positive predictive value (PPV), negative predictive value (NPV), precision, recall, ROC AUC, sensitivity (sens), and specificity (spec). Higher values are represented by blue, while lower values are indicated by white, providing a visual representation of model performance in both training and test sets.

Figure 3A presents the ROC curves for the test set across eight machine learning models: lightGBM, MLP, DT, KNN, RF, SVM, Enet, and XGBoost. Notably, the Enet and MLP models demonstrated the highest AUC performance (AUC = 0.83), significantly outperforming the other six models. Among these, the MLP model was selected for further analysis due to its superior performance across additional evaluation metrics. The Figure 3B also displays the ROC curves for both the training and test sets of the MLP model. Consequently, the interpretability analysis of the best-performing model, MLP, is prioritized in this study. Figure 4 illustrates the comparative performance of the various machine learning models on the training set (A) and the test set (B). Pairwise DeLong tests with FDR correction revealed no significant differences in AUC among RF, XGBoost, and LightGBM (adjusted *p* > 0.28). All ensemble models showed significantly higher AUCs compared to the Decision Tree classifier (adjusted *p* < 0.05) (Supplementary Figure 5). To address potential optimism due to internal SMOTE application, 100 bootstrap iterations were performed. The apparent AUC was 0.938, and the bias-corrected AUC was 0.878, indicating an estimated optimism of 0.060. Calibration analysis revealed initial miscalibration, with a Brier score of 0.136, calibration intercept of −3.116, and slope of 1.389. After applying Platt scaling, calibration substantially improved: Brier score decreased to 0.024, intercept adjusted to 0.000, and slope to 1.000, indicating near-perfect calibration. These results support both the discriminative ability and the probability accuracy of the final calibrated model. Detailed calibration metrics and plots are presented in Supplementary Table 4 and Supplementary Figure 6.

In the sensitivity analysis, drinking and smoking variables were excluded, and models were constructed, yielding a maximum AUROC of 0.77 for both the Enet and KNN models. However, the performance of all models was inferior compared to those that included drinking and smoking as predictors (Supplementary Figure 7). By excluding participants diagnosed within 2 years prior to the survey, the performance of machine learning models remained stable. Among all classifiers, MLP again achieved the highest discrimination with an ROC-AUC of 0.83 (Supplementary Figure 8), similar to that observed in the primary analysis.

### 3.6 Model interpretation

**Figure 5:**
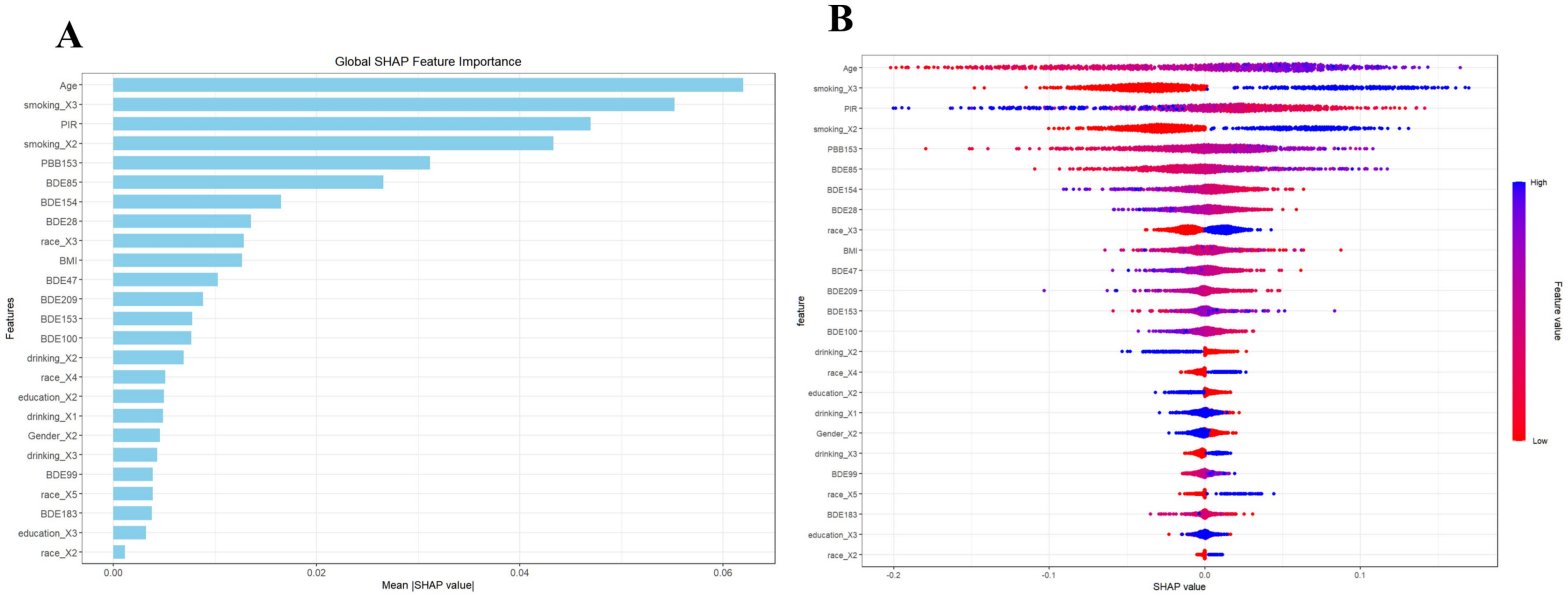
SHAP diagram of MLP model. **(A)** SHAP value ranking of the variables in the model. **(B)** SHAP honeycomb diagram of the MLP model.

SHAP analysis was conducted to evaluate the contribution and importance of each variable in the MLP model’s predictions, as illustrated in Figures 5A,B. The analysis consistently highlighted age as the most significant variable, exhibiting the highest SHAP value and serving as a critical risk factor for emphysema. The most important of the BFRs components was PBB153, which ranked fifth after age in the importance of all variables, while it was consistent with the logistic regression results, and both have a harmful effect on emphysema. The second highest variable with a SHAP value is that now smoker are at greater risk of emphysema, and it is also consistent with the performance improvement of the model after we added the smoking variable.

**Figure 6:**
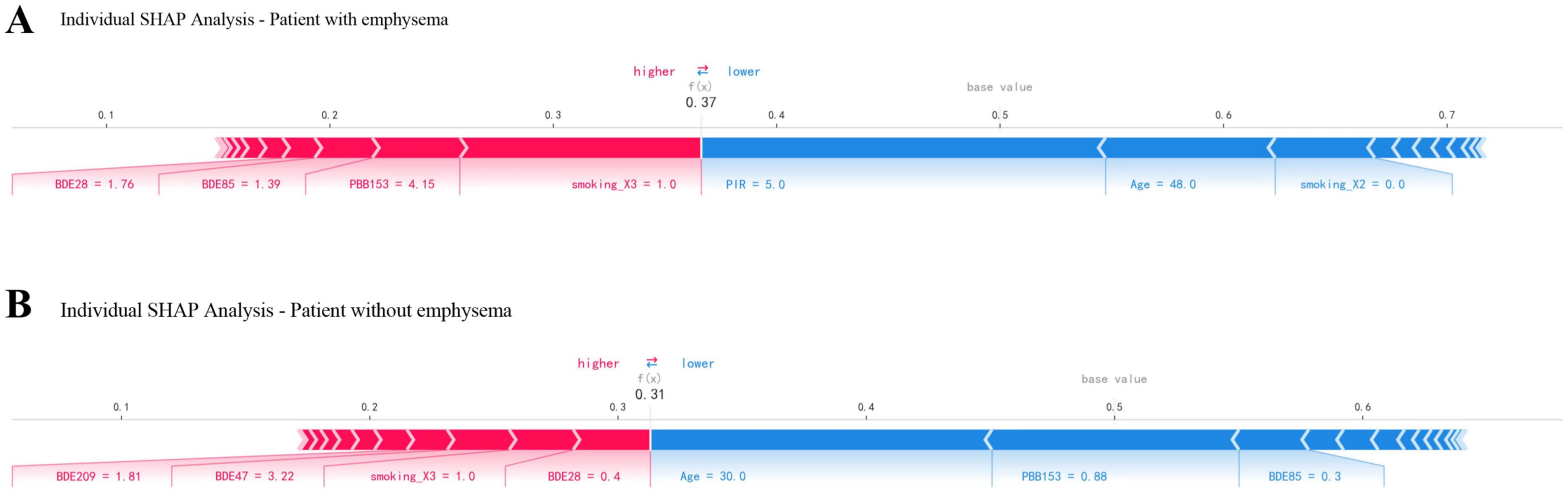
Force plots for 2 patients with and without emphysema.

The Figure 6 presents personalized feature attributions for two representative patients, one with and one without emphysema. The prediction begins from the base value (bias), which represents the average prediction across the training dataset (35). Each feature’s contribution is depicted as an arrow, indicating whether it decreases (negative value) or increases (positive value) the probability of the outcome. The arrows are sorted by their impact on the prediction, with colors representing positive (red) or negative (blue) contributions. The length of each arrow corresponds to the SHAP value for the respective feature. For the patient with emphysema, high levels of BDE28 (1.76), BDE85 (1.39), PBB153 (4.15), and now smoker were major contributors to the elevated risk, counteracted by high PIR (5) and age (48) (Figure 6A). In contrast, for the patient without emphysema, relatively high levels of BDE209 (1.81) and BDE47 (3.22), along with low levels of BDE28 (0.4) and being a current smoker, increased the risk. However, low levels of PBB153 (0.88), BDE85 (0.3), and age (30) reduced the probability of emphysema (Figure 6B).

The PDPs provided a broader understanding of the model’s predictions, highlighting the relationships between emphysema and its predictors, as illustrated in Supplementary Figure 9. The PDP analysis revealed that older age and now smoking status were associated with an increased predicted risk of emphysema. Regarding BFRs, the analysis indicated an upward trend in the predicted probability of emphysema with higher levels of PBB153 and BDE85.

## 4 Discussion

Emphysema, with its rapidly increasing prevalence, has placed a significant burden on individual health and well-being. Environmental chemicals, such as BFRs, which function as endocrine disruptors, have been proposed as overlooked risk factors for COPD (11, 36). This study aimed to investigate the associations between BFR exposure and emphysema and to evaluate the potential predictive value of BFRs for emphysema risk.

Human exposure to various environmental chemicals in the real world is an unavoidable reality. Previous studies have established a significant association between BFR exposure and COPD (36), offering a novel perspective on incorporating BFRs into predictive models for emphysema. However, despite numerous studies on associations, predictive modeling studies that include BFRs as key variables remain limited. Identifying potential environmental biomarkers is crucial for developing high-resolution classifiers for emphysema.

In this study, we developed a ML model using data from the NHANES study (2005–2016) to predict the risk of emphysema in the U.S. population. The model incorporated basic demographic variables and BFR composition data as predictors. Multivariate logistic regression analysis identified significant associations between emphysema risk and several BFRs, including PBB153, BDE28, BDE47, BDE85, BDE99, and BDE153. Additionally, BDE154 and BDE183 were found to be associated with emphysema risk at certain concentrations. WQS analysis further revealed that co-exposure to BFR mixtures significantly increased the risk of emphysema. Among the eight ML models evaluated, the MLP model demonstrated the best predictive performance, achieving an AUC value of 0.83 after cross-validation, indicating its high accuracy in predicting emphysema risk. To further evaluate the robustness of these associations, we conducted a sensitivity analysis by excluding participants who had been diagnosed with emphysema within 2 years of the survey. This adjustment aimed to reduce potential recall bias and mitigate concerns regarding reverse causality. Notably, the results of both logistic regression and machine learning models remained consistent after this exclusion, reinforcing the temporal plausibility and stability of our primary findings. Moreover, given the low prevalence of emphysema and the use of SMOTE for internal oversampling, we conducted optimism-corrected validation using bootstrap analysis. The bias-corrected AUC remained high, indicating strong discriminative ability. Initial probability calibration was poor, but substantially improved after Platt scaling, demonstrating the final model’s clinical applicability in providing reliable risk estimates. SHAP interpretability analysis based on the MLP model highlighted age and PBB153 as the most influential variables, with age contributing the most to the risk of developing emphysema. These findings were corroborated by PDP analysis. Overall, our results suggest that integrating basic demographic information with environmental BFR exposure data has significant potential for enhancing disease risk prediction in future applications.

Previous studies have shown that BFRs are associated with decreased lung function and the development of COPD (36, 37). At the cellular level, BFRs can induce oxidative stress, inflammation, and apoptosis in lung epithelial cells through caspase-dependent mitochondrial pathways (38). BFRs, particularly polybrominated diphenyl ethers (PBDEs), impair the integrity of airway epithelium by decreasing tight junction resistance, reducing zonula occludens-1 expression, and altering mucus production and rheology (39). These effects contribute to barrier dysfunction and increased inflammatory responses in the lungs. Additionally, BFRs have been linked to cardiovascular toxicity and pro-atherosclerotic mechanisms, which may indirectly impact respiratory health (40).

Association and mechanistic studies collectively indicate that the BFRs identified in this study play a significant role in distinguishing emphysema cases. Traditionally, previous research has focused on identifying novel biomarkers or imaging-based predictors for emphysema, often overlooking the potential predictive value of environmental exposures (8). To address this gap, we developed machine learning models to evaluate whether BFRs can reliably predict the risk of emphysema. Given the challenges in accurately understanding ML methodologies and visually interpreting their results, we employed SHAP and PDP analyses in the MLP model to enhance both interpretability and impact. Considering that diseases closely linked to environmental exposures, such as respiratory and cardiovascular diseases, account for approximately one-fourth of all global diseases according to the World Health Organization (WHO), integrating BFRs into predictive models is undoubtedly meaningful. This approach highlights the importance of environmental factors in advancing risk prediction and improving public health strategies.

Our findings offer a novel perspective for researchers in the field of environment and health, contributing to personalized and accurate emphysema risk predictions for individuals at high risk of BFR exposure. However, this study has several limitations. First, emphysema diagnoses were based on self-reported data from questionnaires, which may introduce bias due to recall errors, potentially affecting the accuracy of the risk prediction model. Although we conducted a sensitivity analysis excluding participants diagnosed within 2 years prior to the survey, emphysema could not be reliably defined based on spirometry data in NHANES, as physician-confirmed diagnoses derived from FEV1/FVC measurements were unavailable. Second, serum measurements of BFR concentrations may not fully capture cumulative exposure or tissue-specific levels, as BFRs are known to accumulate in various organs and tissues. Additionally, reliance on a single measurement of BFR levels may not accurately represent long-term exposure patterns. Moreover, within-person variability in serum BFR concentrations may further limit the precision of exposure estimation. Third, more than 80% of the original NHANES sample was excluded due to missing BFR data, as these chemicals were measured only in a one-third subsample. While this missingness was by design, our comparison of weighted characteristics between included and excluded participants revealed systematic differences, suggesting potential selection bias. Fourth, important confounders such as pack-years of smoking, occupational exposure to dust and fumes, passive smoke exposure, and alpha-1-antitrypsin deficiency were not available in the NHANES dataset. Although we calculated E-values indicating the robustness of our findings to potential unmeasured confounding, residual confounding cannot be entirely ruled out. Lastly, given that NHANES is a cross-sectional study, further validation of our prediction model in independent cohort studies is necessary. The lack of imaging data or genetic biomarkers in NHANES also limited our ability to assess whether incorporating BFRs enhances the predictive power of traditional models based on these data types.

## 5 Conclusion

To the best of our knowledge, this is the first study to develop a ML model incorporating BFR exposure data to predict emphysema risk. Using BFR data from NHANES, we constructed and identified the optimal MLP model, which was further interpreted through SHAP and PDP analyses. The model demonstrated excellent predictive accuracy, with PBB153 and age emerging as the most influential variables in the prediction. This study underscores the significant role of BFR exposure in emphysema risk and paves the way for novel approaches to disease prediction, emphasizing the importance of environmental factors in advancing public health research and interventions.

## Data Availability

Publicly available datasets were analyzed in this study. This data can be found at: the National Health and Nutrition Examination Survey (NHANES) https://wwwn.cdc.gov/nchs/nhanes/.

## Footnotes

^1^ www.evalue-calculator.com
