# Supplementary material for: Identifying emphysema risk using brominated flame retardants exposure: a machine learning predictive model based on the SHAP methodology
Source: Front Public Health. 2025 Jun 25;13:1600729. doi: 10.3389/fpubh.2025.1600729 (PMC12238023; doi:10.3389/fpubh.2025.1600729)

Supplemental Material:
eMethods: Model training informations.
eTable 1: Comparison of weighted characteristics between participants included and excluded due to missing BFR measurements.
eTable 2: Multivariate logistic regression analysis of Ln-transformed BFRs for the prevalence of emphysema after excluding participants diagnosed within two years of the survey.
eTable 3: The joint effect of BFRs on the prevalence of emphysema in WQS model.
eTable 4: MLP model discrimination and calibration metrics before and after Platt scaling.
eFigure 1: Distribution of BFRs concentrations before and after Ln-transformed.
eFigure 2: Pearson correlations among the ten BFRs.
eFigure 3: The PCA plots of participants with and without emphysema.
eFigure 4: The WQS model weights of BFRs on the prevalence of emphysema in positive direction.
eFigure 5: Pairwise comparison of model AUCs using DeLong tests with FDR correction.
eFigure 6: Assessment of model discrimination and calibration. (A) Bootstrap distribution of AUC estimates across 100 iterations. (B) Calibration curves comparison.
eFigure 7: AUROC values of the eight ML models after excluding drinking and smoking. (A) AUROC values of the trainingsets. (B) AUROC values of test sets.
eFigure 8: AUROC values of the eight ML models after excluding participants diagnosed within two years of the survey. (A) AUROC values of the trainingsets. (B) AUROC values of test sets.
eFigure 9:Partial dependence plots of the MLP model.

eMethods: Model training informations.
The hyperparameters tuned for different algorithms are given below.

Hyperparameter Search Grids

Decision Tree
- tree_depth: [3, 4, 5, 6, 7]
- min_n: [5, 6, 7, 8, 9, 10]
- cost_complexity: [0.000372, 0.0000893, 0.0338, 0.000172, 0.0151, 0.00488, 0.0114, 0.0000872, 0.00267, 0.00000105]

Random Forest
- mtry: [2, 6, 10]
- trees: [200, 500]
- min_n: [20, 50]

XGBoost
- mtry: [2, 6, 2, 2, 3]
- min_n: [8, 6, 14, 5, 20]
- tree_depth: [3, 3, 1, 1, 2]
- learn_rate: [0.00172, 0.00891, 0.0132, 0.0643, 0.00189]
- loss_reduction: [0.926, 0.692, 0.00177, 0.0349, 0.0148]
- sample_size: [0.981, 0.889, 0.967, 0.948, 0.962]

Elastic Net
- mixture: [0, 0.25, 0.5, 0.75, 1]
- penalty: [0.00001, 0.0000359, 0.000129, 0.000464, 0.00167, 0.00599, 0.0215, 0.0774, 0.278, 1]

Support Vector Machine
- cost: [0.0312, 32]
- rbf_sigma: [0.0001, 0.00316, 0.1]

Single Hidden Layer Neural Network
- hidden_units: [15, 24]
- penalty: [0.001, 1]
- epochs: [50, 150]

LightGBM
- tree_depth: [1, 1, 1, 1, 2]
- trees: [148, 327, 245, 221, 227]
- learn_rate: [0.00324, 0.00823, 0.0274, 0.0740, 0.00841]
- mtry: [3, 6, 7, 5, 3]
- min_n: [7, 7, 5, 5, 8]
- loss_reduction: [0.322, 0.0148, 0.521, 0.0219, 0.163]

K-Nearest Neighbors
neighbors: [3, 5, 7, 9, 11]

Optimal Parameters

Decision Tree
tree_depth: 6
min_n: 6
cost_complexity: 0.00488

Random Forest
mtry: 6
trees: 500
min_n: 20

XGBoost
mtry: 6
min_n: 6
tree_depth: 3
learn_rate: 0.00891
loss_reduction: 0.692
sample_size: 0.889

Elastic Net
mixture: 0
penalty: 0.278

Support Vector Machine
cost: 32
rbf_sigma: 0.1

Single Hidden Layer Neural Network
hidden_units: 15
penalty: 1
epochs: 150

LightGBM
tree_depth: 1
trees: 221
learn_rate: 0.0740
mtry: 5
min_n: 5
loss_reduction: 0.0219

K-Nearest Neighbors
neighbors: 11

eTable 1: Comparison of weighted characteristics between participants included and excluded due to missing BFR measurements.

Participant characteristics	N (%) or Mean (SD)			
	Overall	Include BFRs	Exclude BFRs	P-value
N	1709384753	346236391	1363148362	
Age（years）	37.37 (22.41)	47.20 (16.75)	34.88 (22.98)	<0.001
Gender				0.26
Male	837869283.0 (49.0)	167776306.0 (48.5)	670092977.0 (49.2)	
Female	871515470.0 (51.0)	178460085.0 (51.5)	693055385.0 (50.8)	
Race or ethnicity				<0.001
Mexican American	171394365.0 (10.0)	27628478.0 (8.0)	143765887.0 (10.5)	
Other Hispanic	97748660.0 (5.7)	17802755.0 (5.1)	79945905.0 (5.9)	
Non-Hispanic White	1103688162.0 (64.6)	240825974.0 (69.6)	862862188.0 (63.3)	
Non-Hispanic Black	207208793.0 (12.1)	37006004.0 (10.7)	170202789.0 (12.5)	
Other Race - Including Multi-Racial	129344773.0 (7.6)	22973180.0 (6.6)	106371593.0 (7.8)	
Education level				<0.001
Less than high school	481872692.0 (28.2)	53882914.0 (15.6)	427989778.0 (31.4)	
High School or Equivalent	302239167.0 (17.7)	79217077.0 (22.9)	223022090.0 (16.4)	
College or above	778261794.0 (45.5)	213136400.0 (61.6)	565125394.0 (41.5)	
Missing	147011100.0 (8.6)	0.0 (0.0)	147011100.0 (10.8)	
PIR	2.85 (1.66)	3.04 (1.63)	2.80 (1.66)	<0.001
BMI(kg/m2)	26.79 (7.56)	28.86 (6.62)	26.23 (7.70)	<0.001
Drinking status				<0.001
Never	141716471.0 (8.3)	36846437.0 (10.6)	104870034.0 (7.7)	
Mild，moderate	619556559.0 (36.2)	182200689.0 (52.6)	437355870.0 (32.1)	
Heavy	255502281.0 (14.9)	74697209.0 (21.6)	180805072.0 (13.3)	
Former	183884671.0 (10.8)	52492056.0 (15.2)	131392615.0 (9.6)	
Missing	508724771.0 (29.8)	0.0 (0.0)	508724771.0 (37.3)	
Smoking status				<0.001
Never	705291761.0 (41.3)	189359815.0 (54.7)	515931946.0 (37.8)	
Former	312504305.0 (18.3)	86473127.0 (25.0)	226031178.0 (16.6)	
Now	264933034.0 (15.5)	70403449.0 (20.3)	194529585.0 (14.3)	
Missing	426655653.0 (25.0)	0.0 (0.0)	426655653.0 (31.3)	

Data are n (%), mean (SD). PIR: Poverty to income ratio; BMI: Body mass index.

eTable 2:Multivariate logistic regression analysis of Ln-transformed BFRs for the prevalence of emphysema after excluding participants diagnosed within two years of the survey.

	Model I			Model II		
Variable	OR (95%CI)	P value	E-value (CI)	OR (95%CI)	P value	E-value (CI)
LnPBB153	**1.84 (1.59-2.14)**	**<0.001**	3.08 (2.56)	**1.32 (1.09-1.6)**	**0.005**	1.97 (1.4)
Q1	Reference			Reference		
Q2	**4.02 (1.13-14.26)**	**0.031**	7.5 (1.51)	1.57 (0.42-5.86)	0.502	2.52 (1)
Q3	**12.87 (3.97-41.76)**	**<0.001**	25.23 (7.4)	2.96 (0.83-10.6)	0.095	5.37 (1)
Q4	**24.05 (7.56-76.52)**	**<0.001**	47.59 (14.6)	**4.45 (1.24-15.94)**	**0.022**	8.37 (1.79)
P for trend	<0.001			<0.001		
LnBDE209	0.94 (0.66-1.34)	0.737	1.32 (1)	0.84 (0.62-1.16)	0.297	1.67 (1)
Q1	Reference			Reference		
Q2	1.04 (0.6-1.8)	0.888	1.24 (1)	1.1 (0.62-1.96)	0.745	1.43 (1)
Q3	1.5 (0.94-2.39)	0.092	2.37 (1)	1.4 (0.86-2.3)	0.177	2.15 (1)
Q4	0.78 (0.44-1.37)	0.386	1.88 (1)	0.77 (0.42-1.39)	0.384	1.92 (1)
P for trend	0.863			0.744		
LnBDE28	**1.47 (1.11-1.94)**	**0.007**	2.3 (1.46)	0.97 (0.74-1.28)	0.85	1.21 (1)
Q1	Reference			Reference		
Q2	1.74 (0.99-3.07)	0.055	2.92 (1.31)	1.23 (0.68-2.23)	0.487	1.76 (1)
Q3	1.74 (0.99-3.07)	0.056	2.87 (1)	1.02 (0.57-1.84)	0.946	1.16 (1)
Q4	**2.01 (1.16-3.5)**	**0.013**	3.43 (1.59)	0.92 (0.51-1.66)	0.783	1.39 (1)
P for trend	0.022			0.497		
LnBDE47	1.24 (0.97-1.6)	0.086	1.79 (1)	1.01 (0.8-1.27)	0.962	1.11 (1)
Q1	Reference			Reference		
Q2	1.04 (0.6-1.79)	0.887	1.24 (1)	0.75 (0.43-1.33)	0.327	2 (1)
Q3	1.23 (0.73-2.07)	0.436	1.76 (1)	0.88 (0.51-1.51)	0.634	1.53 (1)
Q4	1.46 (0.89-2.42)	0.137	2.28 (1)	0.81 (0.47-1.38)	0.438	1.77 (1)
P for trend	0.101			0.632		
LnBDE85	1.25 (1-1.56)	0.053	1.81 (1)	1.1 (0.9-1.36)	0.352	1.43 (1)
Q1	Reference			Reference		
Q2	1.37 (0.79-2.38)	0.268	2.08 (1)	1.05 (0.59-1.87)	0.865	1.28 (1)
Q3	1.5 (0.87-2.59)	0.142	2.37 (1)	1.16 (0.66-2.04)	0.611	1.59 (1)
Q4	**1.73 (1.02-2.94)**	**0.042**	2.85 (1.16)	1.14 (0.65-1.99)	0.647	1.54 (1)
P for trend	0.041			0.386		
LnBDE99	1.18 (0.95-1.46)	0.135	1.64 (1)	1.03 (0.84-1.25)	0.793	1.21 (1)
Q1	Reference			Reference		
Q2	0.63 (0.35-1.12)	0.117	2.55 (1)	0.54 (0.3-0.99)	0.047	3.11 (1.11)
Q3	1.27 (0.79-2.06)	0.325	1.86 (1)	1.02 (0.61-1.69)	0.938	1.16 (1)
Q4	1.2 (0.74-1.96)	0.463	1.69 (1)	0.77 (0.46-1.3)	0.328	1.92 (1)
P for trend	0.135			0.838		
LnBDE100	1.13 (0.88-1.45)	0.346	1.51 (1)	1 (0.79-1.25)	0.979	1 (1)
Q1	Reference			Reference		
Q2	0.9 (0.53-1.53)	0.684	1.46 (1)	0.7 (0.4-1.22)	0.213	2.21(1)
Q3	1.21 (0.74-1.98)	0.455	1.71 (1)	1.05 (0.62-1.77)	0.865	1.28 (1)
Q4	1.14 (0.69-1.88)	0.616	1.54 (1)	0.77 (0.45-1.31)	0.327	1.92 (1)
P for trend	0.4			0.655		
LnBDE153	**1.37 (1.07-1.75)**	**0.013**	2.08 (1.34)	1.13 (0.9-1.42)	0.283	1.51 (1)
Q1	Reference			Reference		
Q2	1.32 (0.78-2.23)	0.294	1.97 (1)	1.3 (0.75-2.26)	0.343	1.92 (1)
Q3	0.88 (0.49-1.56)	0.661	1.53 (1)	0.75 (0.41-1.37)	0.355	2 (1)
Q4	**1.73 (1.05-2.84)**	**0.031**	2.85 (1.28)	1.33 (0.78-2.26)	0.294	1.99 (1)
P for trend	0.083			0.597		
LnBDE154	1.16 (0.92-1.46)	0.211	1.59 (1）	0.98 (0.79-1.21)	0.832	1.16 (1)
Q1	Reference			Reference		
Q2	1.29 (0.75-2.21)	0.352	1.9 (1)	0.89 (0.51-1.55)	0.673	1.5 (1)
Q3	1.42 (0.84-2.41)	0.188	2.19 (1)	1.04 (0.6-1.81)	0.877	1.24 (1)
Q4	1.41 (0.83-2.39)	0.201	2.17 (1)	0.84 (0.48-1.47)	0.54	1.56 (1)
P for trend	0.191			0.684		
LnBDE183	1 (0.73-1.36)	0.976	1 (1)	0.87 (0.64-1.19)	0.386	1.56 (1)
Q1	Reference			Reference		
Q2	1.53 (0.86-2.73)	0.148	2.43 (1)	1.34 (0.73-2.46)	0.339	2.01 (1)
Q3	**2.1 (1.19-3.7)**	**0.01**	3.62 (1.67)	1.62 (0.9-2.93)	0.109	2.62 (1)
Q4	1.26 (0.68-2.33)	0.459	1.83 (1)	1.03 (0.53-1.99)	0.934	1.21 (1)
P for trend	0.354			0.893		

Model 1: no covariates were adjusted. Model 2: adjusted for all covariates.

eTable 3:The joint effect of BFRs on the prevalence of emphysema in WQS model.

Outcome	OR	95% CI	P-value
Positive			
Model I	2.28	1.80-2.89	<0.001
Model II	1.51	1.11-2.06	0.008
Negative			
Model I	0.93	0.75-1.16	0.522
Model II	0.86	0.65-1.14	0.291

Model I adjusted for none, while Model II adjusted for all covariates.

eTable 4: MLP model discrimination and calibration metrics before and after Platt scaling.

Metric	Original.Model	Platt.Scaling	Quantile.Calibration	Improvement (Platt)
Brier Score	0.1357	0.024	0.0239	82.30%
Calibration Intercept	-3.1157	0	0.046	100%
Calibration Slope	1.3885	1	1.0167	100%
Calibration Error	0.3216	0.0058	0.0001	98.20%
Discrimination (AUC)	0.878	0.878	0.878	Unchanged

eFigure 1: Distribution of BFRs concentrations before and after Ln-transformed.

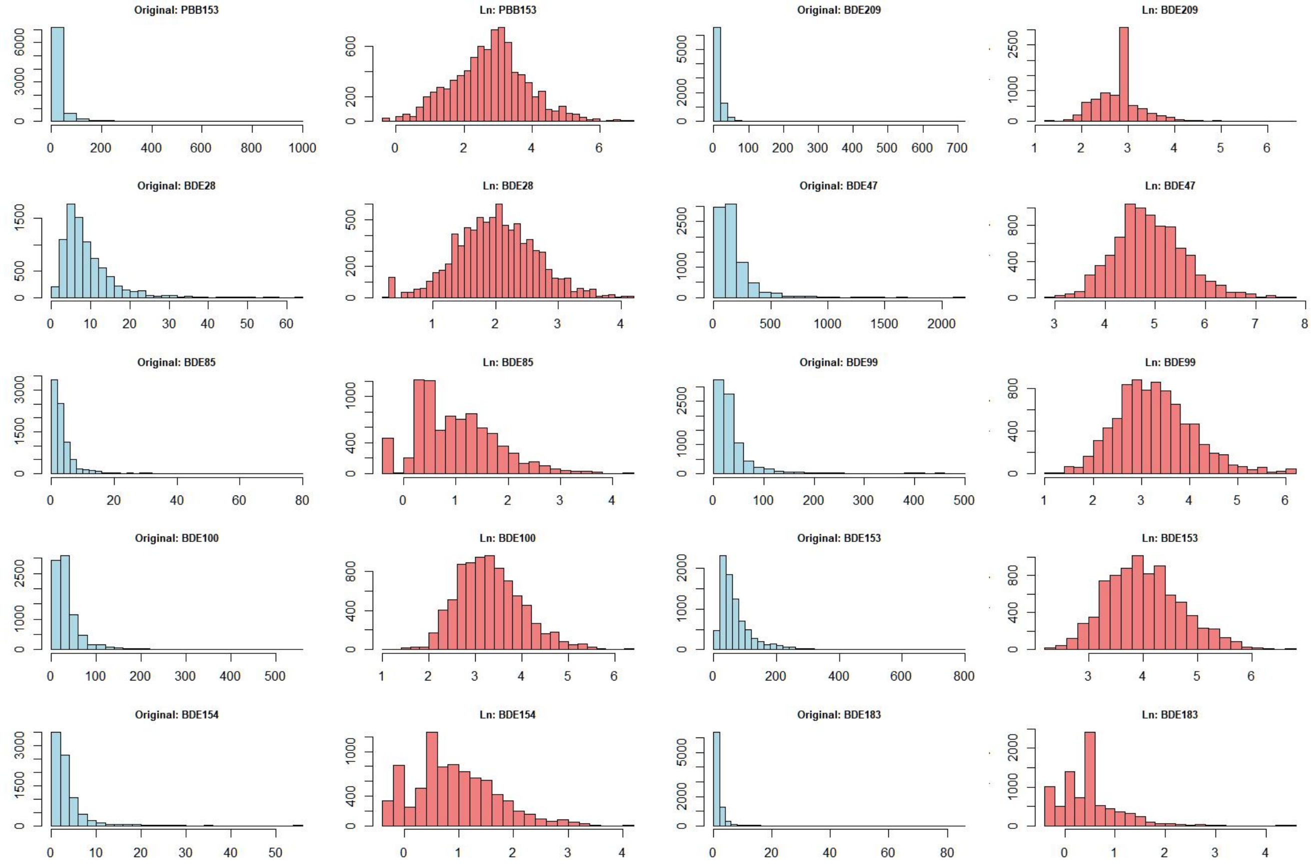

eFigure 2:Pearson correlations among the ten BFRs.

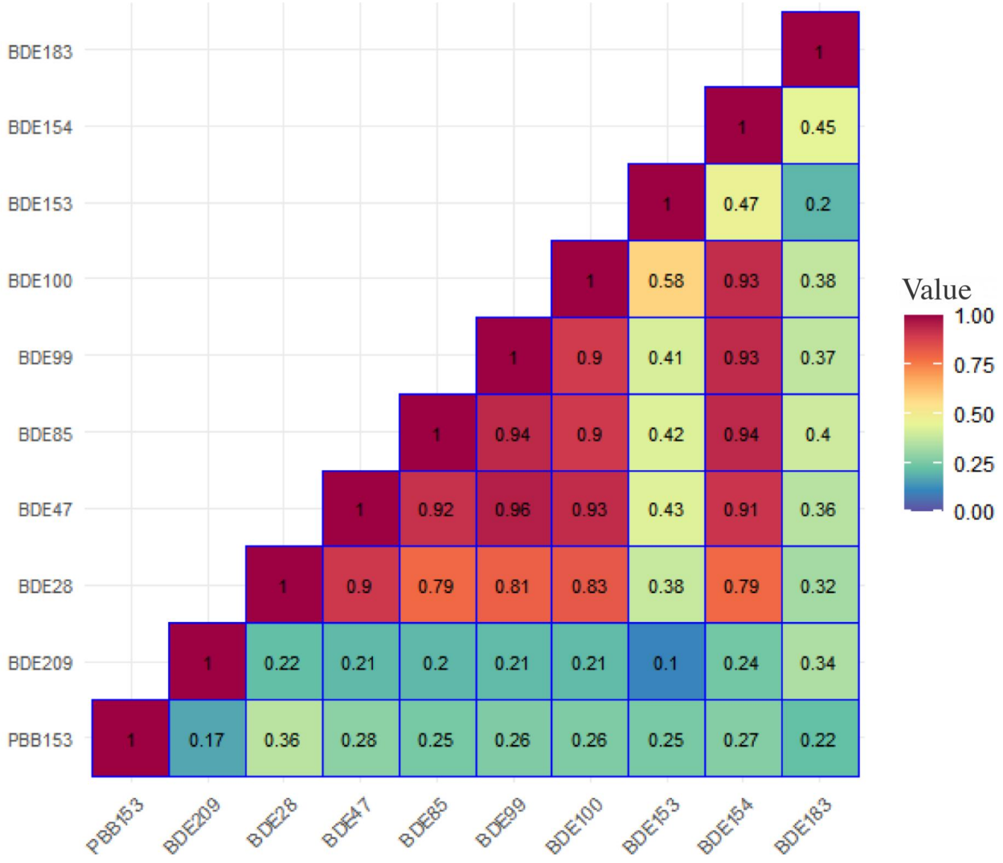

eFigure 3:The PCA plots of participants with and without emphysema.

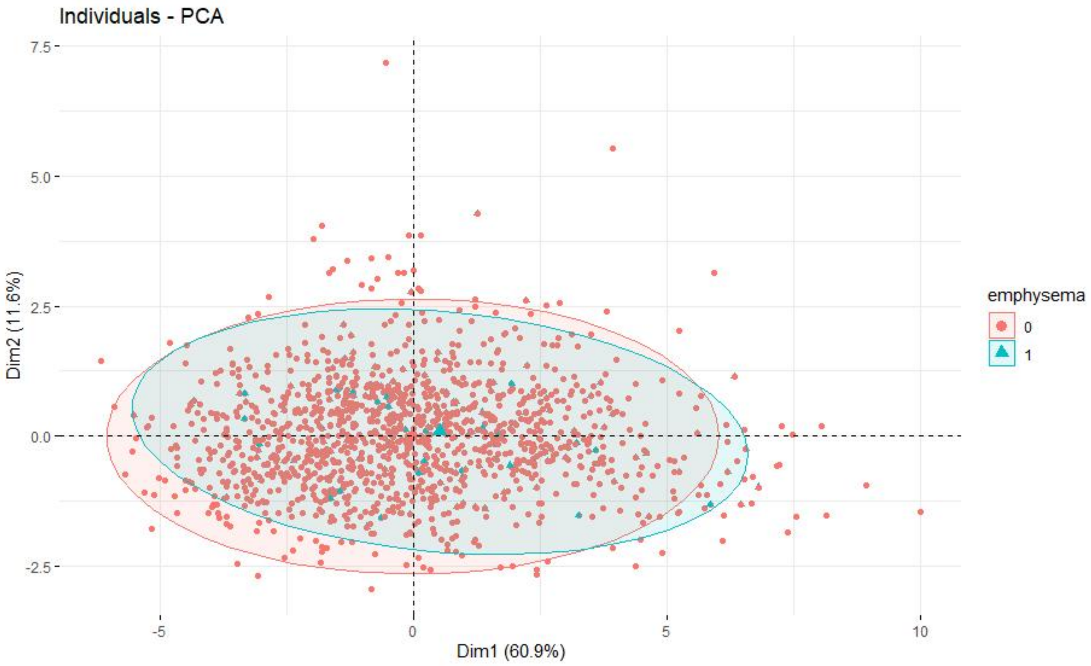

eFigure 4: The WQS model weights of BFRs on the prevalence of emphysema in positive direction.

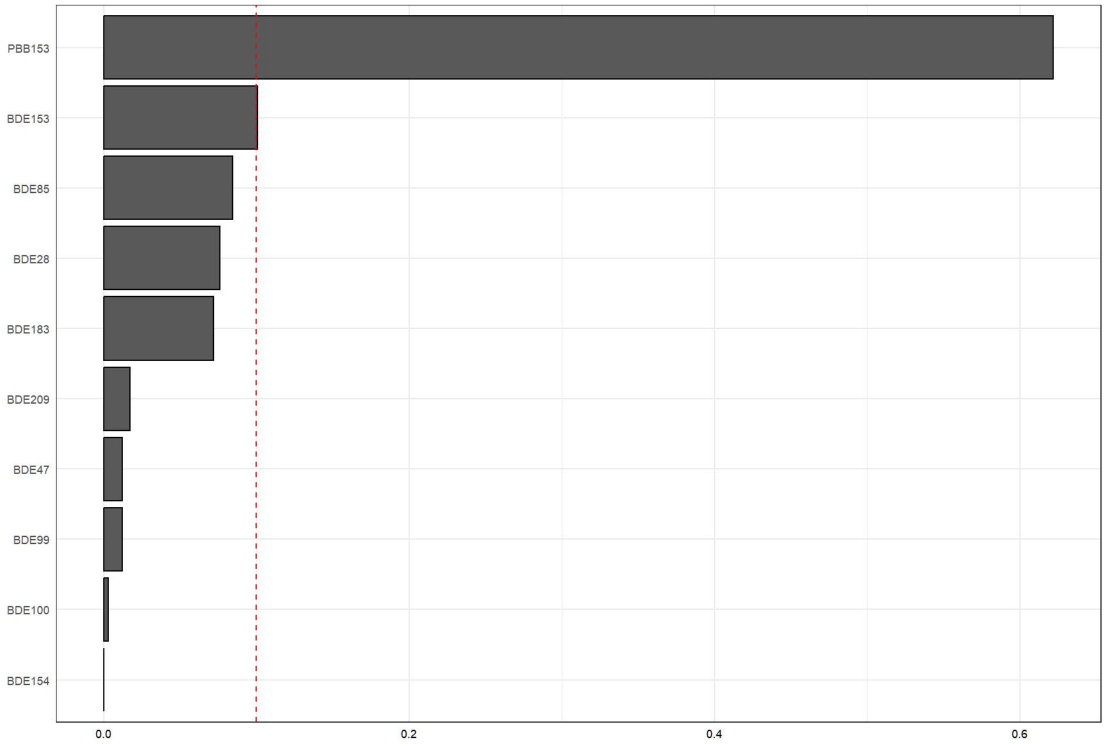

eFigure 5: Pairwise comparison of model AUCs using DeLong tests with FDR correction. .

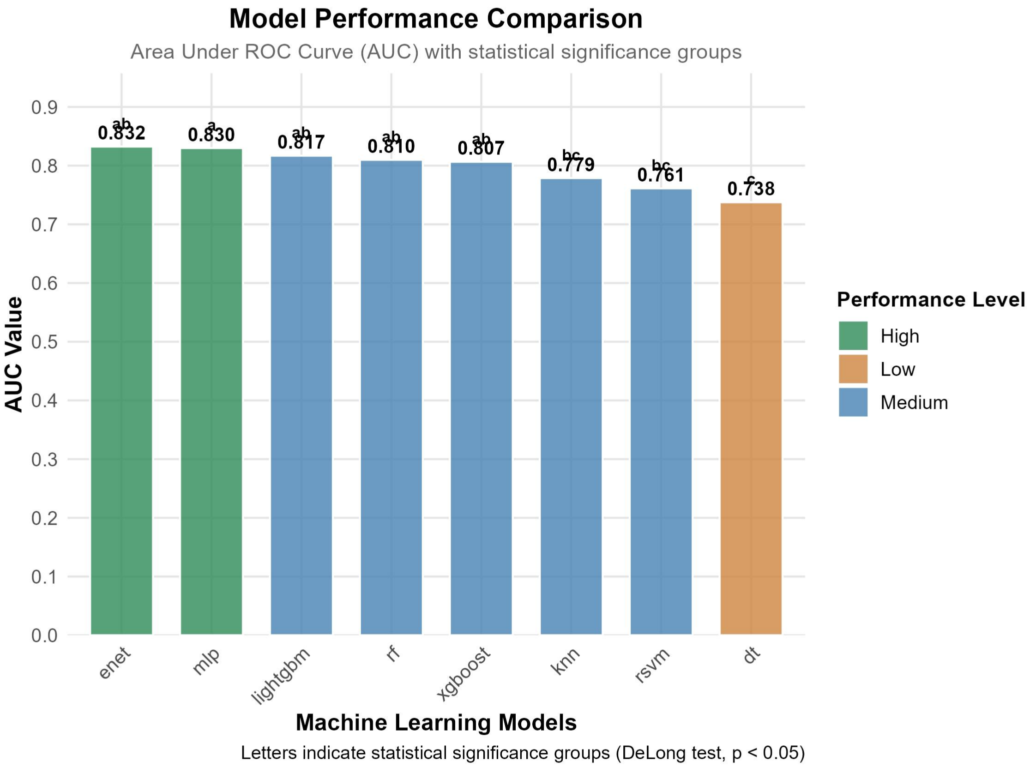

Bars represent the mean AUC of each machine learning model. Letters above the bars denote statistical groupings based on pairwise DeLong tests (FDR-adjusted p < 0.05): models sharing the same letter do not differ significantly.

eFigure 6: Assessment of model discrimination and calibration. (A) Bootstrap distribution of AUC estimates across 100 iterations. (B) Calibration curves comparison.

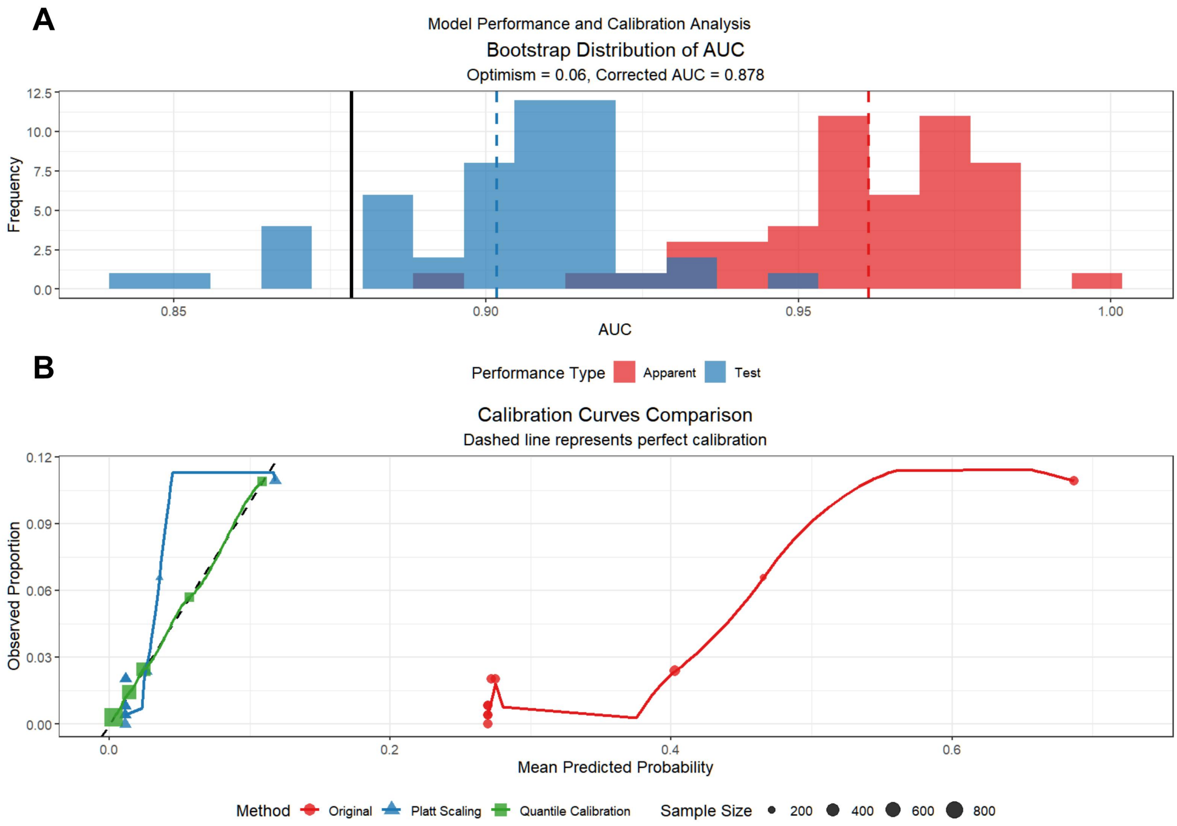

eFigure 7: AUROC values of the eight ML models after excluding drinking and smoking. (A) AUROC values of the trainingsets. (B) AUROC values of test sets.

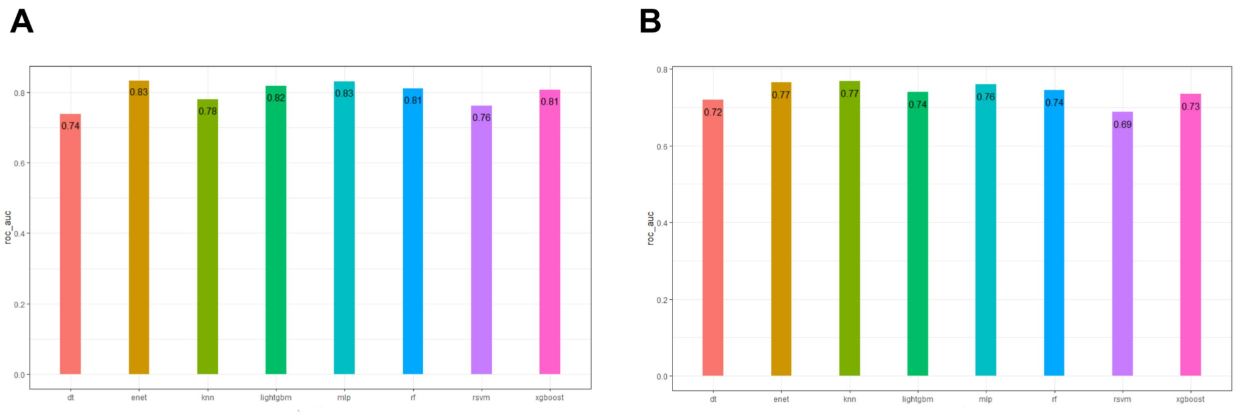

eFigure 8: AUROC values of the eight ML models after excluding participants diagnosed within two years of the survey. (A) AUROC values of the training sets. (B) AUROC values of test sets.

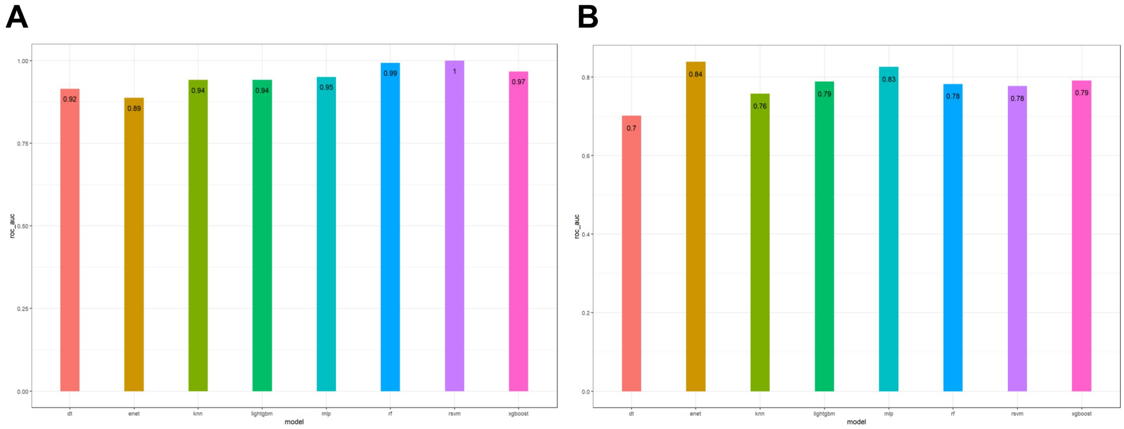

eFigure 9:Partial dependence plots of the MLP model.

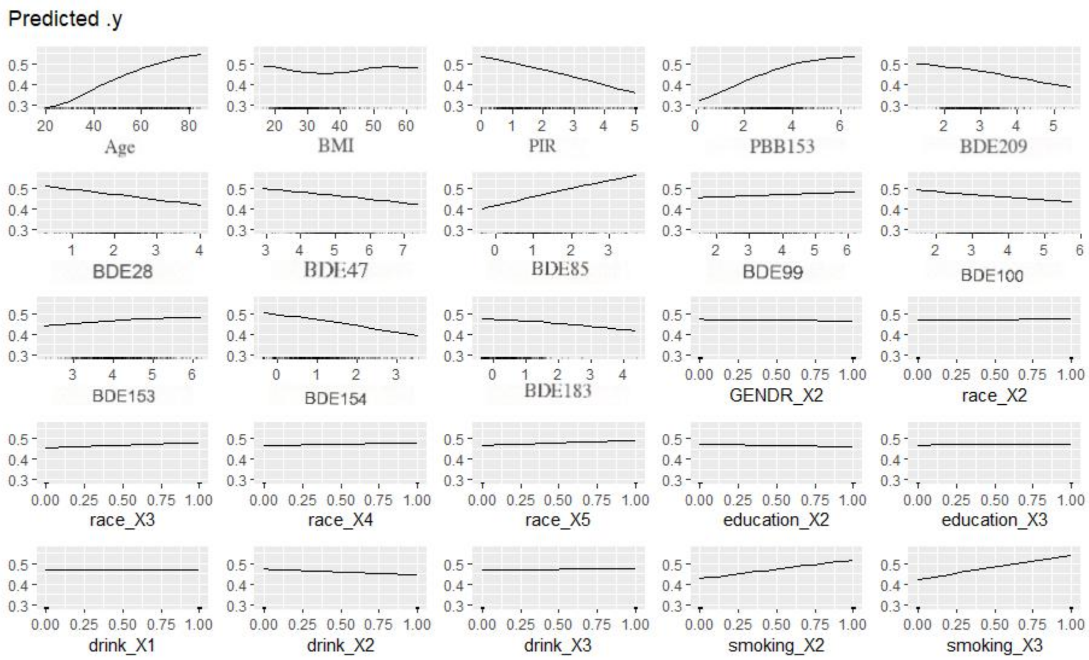

Supplement: Supplementary file 1 [file Data_Sheet_1.pdf]
